# Understanding and barriers to formation Professional Identity among current and recent graduates of nurses and midwifery in two universities in a low resource setting: A qualitative study

**DOI:** 10.21203/rs.3.rs-3423723/v1

**Published:** 2023-10-13

**Authors:** Scovia Nalugo Mbalinda, Josephine Nambi Najjuma, Aloysius Gonzaga Mubuuke, Livingstone Kamoga, David Musoke

**Affiliations:** Makerere University; Mbarara University of Science and Technology; Makerere University; Makerere University; Makerere University

**Keywords:** Professional Identity, Nurses, Midwives, Barriers, Uganda

## Abstract

**Introduction:**

Professional identity (PI) in nursing is a sense of oneself and one’s relationship with others that is influenced by characteristics, norms, and values of the nursing discipline, resulting in an individual thinking, acting, and feeling like a nurse. Therefore, transformative educational approaches that include professional judgement, reasoning, critical self-evaluation and a sense of accountability are required to foster professional identity. We explored the understanding and barriers to professional identity formation among recent graduates and nursing students in Uganda.

**Methods:**

A qualitative research design was used to collect data from student Nurses and Midwives from Makerere University, Mbarara University and recent graduates attending their internship training at Mulago National and Mbarara Regional Referral hospitals. Thematic analysis was used to analyse the data.

**Results:**

The participants who reported understanding of PI in nursing and midwifery mentioned that these are principles, characteristics and values, competencies, ethics and code of conduct, sense of belonging and professionalism that define the nursing profession and practice. Barriers to the formation of PI were provided under two themes education and health service delivery. Regarding education (nursing educators not working in clinical settings and inadequate clinical mentoring). Under health service delivery:(high workload, lack of interprofessional collaboration, many different professional groups, no clear scope of practice for the different professional careers, Low esteem among nurses and midwives, media and lack of policy implementation).

**Conclusion and recommendation:**

Participants were knowledgeable about professional identity in nursing/midwifery. They faced several challenges and barriers in professional identity formation during their training and internship. We recommend a need to streamline the scope of practice and enhance clinical mentorship and engagement of Leadership in Nursing in developing PI among students.

## Introduction

Rapid changes in nursing education, regulation, and practice require a “clear understanding of the professional identity (PI) of a nurse so that their unique contribution to health improvement is recognised”[1].In addition, nurses with a strong PI may experience higher job satisfaction, which impacts retention[2, 3]. According to Wei, Zhou, Hu, Zhou, and Chen (2021), PI positively impacts building a positive self-image, professional satisfaction, a sense of belonging, and recognition of an individual’s professional competence [4]. Therefore, nurses must be aware of the importance and value of professional identity [5]. Professional identity can be an individual construct of self or a collective representation of a profession [2]. Brown et al. (2018) reported that nursing students’ perceptions of their professional identity include paying attention to the role, performing different roles, connecting with others, and caring for themselves. The International Society for Professional Identity in Nursing (ISPIN), founded in the USA, adopted Godfrey and Young’s (2020) definition of professional identity in nursing as “a sense of self and concerning others that is influenced by characteristics, norms, and values of the nursing discipline and results in the individual thinking, acting, and feeling like a nurse”[6]. The four domains derived from this definition include values and ethics, knowledge, leadership and professional behaviour (International Society for Professional Identity in Nursing [ISPIN], 2020). The complex healthcare environment requires nurses to take a leadership role to provide safe and quality care. Similarly, Simmonds et al. (2020) reported that beliefs and values, nursing knowledge and skills, and professional roles in nursing are components of professional identity formation. However, these authors added two more components: belongingness and personality [7].

PI is developed over time through different strategies, and nursing school is one of the avenues where PI is developed. The development of a professional identity is a lifelong process that begins before enrolment in a nursing program and is based on the preconceived skills, attributes, behaviour, culture and ideology of the intended profession [2]. Students form their professional identities through engagement and reflection on multiple experiences that lead them to embrace the profession’s history, characteristics, norms and values [8, 9]. Therefore, transformative educational approaches that incorporate professional judgement, reasoning, critical self-evaluation and a sense of accountability are necessary to promote professional identity development [7]. Professional identity development in nursing is dynamic and influenced by multidimensional factors [10], leading to deeper insight into and commitment to professional practice. Positive clinical learning experiences and relationships facilitate the development of a professional self-concept). Students who felt that nursing educators promoted professional identity formation during clinical experiences felt empowered to think like a nurse [8]. In addition to positive perceptions of the clinical learning environment, Wu, Palmer, and Sha (2020) found that a clinical experience of more than eight months was a dominant factor positively associated with professional identity [3]. In their review paper, Mao, Lu, Lin and He (2021) concluded that personal, family, institutional and social factors influence the development of Chinese nursing students’ professional identity. According to Rasmussen et al. (2021), self, role, patient care, environment, healthcare team, and perception of nursing influence the professional identity of registered nurses. Dynamic forces influencing professional identity are professional development and time [11].

Student nurses, however, are relatively powerless in the healthcare hierarchy, which might affect their PI. At Mbarara University of Science and Technology (MUST) and Makerere University (MAK), nurses with higher education teach trainee nurses in the classroom. However, they are taught at the bedside by a mix of nurses and professionals. The latter does not contribute to developing the professional identity of student nurses and midwives. Evidence suggests that a negative image of nursing and midwifery does not promote these professions as attractive career options [12]. Furthermore, few studies document how nursing and midwifery are perceived in East Africa; where such studies exist, they are country-specific [12]. Therefore, the study will explore the understanding and barriers to professional identity among current students and recent graduates in Uganda.

## Methods

### Study design and setting

This descriptive qualitative study assessed the understanding of Professional Identity and barriers to developing professional identity among current and recent midwives and nursing graduates.

The study was conducted among current degree students of midwives and nursing at Mbarara University of Science and Technology (MUST) and Makerere University. The two universities are the oldest in Uganda and produce the largest number of nursing and midwifery graduates in the country. They are both government universities. We also included recent midwives and nursing graduates practising nursing as part of their one-year mandatory training (internship) at Mulago National Referral Hospital and Mbarara Regional Referral Hospital. Both hospitals receive interns from any of Uganda’s 9-degree nurses training institutions. MNRH is found in Kampala, the capital city, and serves as a national referral for all the hospitals in the country. It provides a range of services. MRRH is found in southwestern Uganda and serves about 14 districts and the neighboring countries in southwestern Uganda.

### Study participants and recruitment

The participants of the study were nursing and midwifery students at Makerere University and Mbarara University of Science and Technology, and recent graduates are attending their internship placement at Mulago National Referral Hospital and Mbarara Regional Referral Hospital. W purposively planned to include male and female participants willing to participate in the study and provided written informed consent. Also, the two universities and hospitals were purposively selected because they produced or received many nurses and midwives. We excluded masters of nursing and midwifery students since this group might have developed a professional identity.

### Study procedure:

Focus Group Discussions were conducted by a trained research assistant with a nursing background, and a trained assistant also took the field notes with a nursing background. Data was collected between March 2023 to August 2023. Each discussion lasted about 45 to 60 minutes. All participants were assured of their confidentiality. Written informed consent was sought, and the participants were told about their right to participate and withdraw at any time without penalty.

### Data collection:

Data was collected from student Nurses and Midwives from Makerere University and Mbarara University, and recent graduates from Mulago National and Mbarara regional referral Hospitals were recruited into this study. Prior to data collection, written informed consent was sought from the participants. Consent was explained in English. After obtaining informed consent, each participant responded to a brief questionnaire.

The FGD guide was developed by the lead researcher (SNM) in consultation with a nurse and qualitative researcher (JNN) and was later reviewed by a senior qualitative researcher (JRM). The questionnaire sought information on their age, gender, occupational status, marital status, and the reason for entering a nursing program. The demographic questionnaire provided context for the participants. We conducted 6 Focus group discussions amongst recent graduates and students of nursing/midwifery. All the interviews were audio recorded, and notes were taken upon consent of the participants. We discussed the definition of Professional Identity in Nursing. We required them to describe challenges and barriers to professional identity formation. Identify nurse educator practices that will foster Professional Identity in Nursing.

Participants were also encouraged to share any additional information they felt was important to the conversation. They were informed that in case distress occurred to them during the interview, the meeting was to be stopped and continued when the participant could go on with it. These interviews were audio-recorded and lasted between 40 to 60 minutes long.

### Data management and analysis

All Focus group discussions were recorded. Data from the interviews consisted of transcripts of the audio-recorded sessions and debriefing notes and memos from the study team members facilitating the interviews. Recorded interviews were transcribed verbatim. Open coding of the data (Level I) began the data analysis, which involved line-by-line analysis of the transcribed data from the interviews to identify the processes and contextual factors in the data. These factors or substantive codes were compared with other data and assigned categories (Level II). Categories composed of coded data that appeared to form patterns or exhibited similar information. The categories were then compared to others to ensure they were mutually exclusive. Categories were then reduced by comparing them to each other to determine how they fit in a higher-order category. The number of categories were reduced to identify the primary social processes or core variables that explained the social scene (Level III). We conceptualised the relationships among the three code levels by developing more theoretical Level III codes [13].

### Ethical Consideration

All methods were carried out according to the relevant guidelines and regulations of the Declaration of Helsinki (DoH-Oct2008). Before data collection, ethical approval was obtained from Makerere University College of Health Sciences, the School of Health Sciences Institutional Review Board (IRB) (MAKSHSREC 2022 − 415), and the Uganda National Council for Science and Technology (UNCST)(HS2712ES) Administrative clearances were obtained from the hospitals’ and Universities’ administration where we conducted the study. Informed consent was obtained from the participants. The research team informed the participants at all levels about the survey and requested their voluntary participation. All respondents were assured of confidentiality concerning the matters under discussion as the interviews were conducted in special rooms. Audiotapes and notes didn’t contain participants’ identifiers and were kept in a locked file cabinet when not used.

## Results

A total of 33 students and 26 recent graduates, 40 females and 19 males, aged 20 to 51 years (median 25 years), participated in six (6) focus group discussions FGDs (n = 59).

### Understanding of Professional Identity in Nursing

The participants were asked about their understanding of PI in nursing, and the following themes emerged;

#### Principles, characteristics and values,

The participants defined professional identity as principles, characteristics and values that govern the professional. They emphasised that anyone in Nursing should have these values and principles, as stated in the quotations below.

“To me, professional identity, I can define it like the code of conduct that governs a given profession and maybe the principles that identify that profession. Or, in another way, the principles that are in each profession that anyone can use to know that this is maybe a profession of nurses (P02 FGD2 student). Another participant added that;

I perceive professional identity as the key characteristics of the profession that make it different from the rest of other professions, like, for instance, your scope of practice, what you are supposed to do within your profession that doesn’t cross maybe in others (P01 fgd1 recent graduate).

### Competencies

The participants also mentioned that you must have competencies and training to practice as a nurse or midwife. They mentioned that a nurse and midwife must demonstrate professional behaviour through their appearance and actions, as portrayed below;

‘Features that are not common to other groups of people that are unique to us as nurses and midwives like uniform, different cadres, the way they put on. So, something that’s making us unique’ (P03 FGD2 recent graduate).

‘You must know what you are doing on the ward; the care you provide must meet the standards of your training and must meet the quality of care expected by the patients’ (P04 FGD2 student nurse)

### Ethics and code of conduct

The participants also mention that professional identity has to do with the ethics and code of conduct of as nurse. There are some codes of conduct outlined by the leadership body that every nurse must observe and abide by them.

I also understand professional identity as the guidelines and ethics of the profession. And how maybe we marry together our personal values and beliefs with those of the profession at the end of the day, and the picture that comes out is the professional identity (P02 FGD1 student).

### Sense of belonging

The participants further mentioned that professional identity is related to a sense of belonging and professionalism that defines the nursing profession and practice, like the role played by registration councils and associations.

To me, I understand it as a sense of belonging to a certain profession, and to say that I am proud to be part of that profession (P03 FGD1 recent graduate)

### Barriers to professional identity formation

The participants were asked about the barriers to the formation of PI, and themes identified include the education delivery (Nursing educators not working in the clinical area and inadequate clinical mentoring), ……… and health service delivery theme (high workload, lack of interprofessional collaboration, many carders in Nursing, no clear scope of practice for each carder, Low esteem among nurses and midwives, media and lack of policy implementation.

### Nursing educators/ Faculty not working in a clinical area

The participants acknowledged that nurse educators teach them in class; however, most of them don’t follow them up in the clinical area. It is expected that clinical health workers should mentor them.

Someone teaching you may never be the same person teaching on the ward or even never be attached to any hospital practicing as a licensed nurse, and some of the clinical people might not have up-to-date information, and there may be a misalignment of information (P01 FGD2 recent graduate).

### Inadequate clinical mentoring

Inadequate mentorship can affect professional identity formation by providing limited learning opportunities, lack of emotional support, and lack of guidance. The participants acknowledged that they lack clinical mentorship by the faculty in the clinical areas.

what I have really observed our frustrations start right away from training schools even before someone is out, and this comes in line with the teaching methods that are employed because what I have noticed most of gov’t institutions the teaching methods are not good. When they teach you in class then they would send you on the ward, there’s no tutor or a nurse who taught you in class has followed you on ward to really whether find you are applying what they taught you. students being neglected. (P02 FGD3, Student nurse).

### High workload

During the clinical rotation, it’s expected that the clinical preceptors should have time to work with the students; however, the clinical people have a lot of patients to look after, which might affect helping students in professional identity formation. This is highlighted in the participants quotes below,

“What frustrates me is the high number of patients, who are too many for one nurse, and it becomes a problem for them to pull out all that professionalism that you need to make everyone receive care as they are supposed to…” (P02 FGD2 recent graduate).

“I was in the hospital, and we were taking vitals………….we couldn’t take the vitals of every patient, we had to stop and do something else,………..So sometimes the circumstance on the different wards affects the professional identity formation, because you have a ward full of patients, and you have the desire to do the right thing, but because of circumstances….and being overloaded, you’ll end up not doing what you’d have done, according to your training and profession “(PO7 FGD3 student).

### Lack of intra and interprofessional collaboration

To facilitate professional identity, there is a need for interprofessional identity on the ward so that everyone on the ward can clearly know their role and the value they add to the team.

..the team we work with in the hospital also greatly impacts our professional identity. Like the medical doctors, the way they interact with the different nurses on the ward and the picture that we sometimes perceive as students is that really there is that lack of teamwork, so they tend to undermine a lot of nurse’s capabilities and maybe what they can do (PO2 FGD3 student).

Another barrier is lack of cooperation; if nursing staff see the intern is out there working on the ward, they really don’t mind coming for the duty because they think the intern will do the work, whereas we are still learning from them(P02 FGD2 Recent graduate)

In most cases, you find that you tend to work in isolation when you are on the ward. I remember when we were students in a certain regional Referral hospital we would end up just teaching ourselves or end up being at the ‘ ‘Doctor’s ward round, and nurses will never come in. I think nurses just see students as people to waive them off from their labour. Maybe they would want students to work with them but not necessarily teach them (P01 FGD2 Recent graduate).

### Many carders in Nursing

The participants mentioned that there are a number of carders in Nursing. When you go to the ward, some feel threatened because they feel that you will get a higher qualification than them. This affects their mentorship in professional identity.

There are many carders in nursing, from certificates to masters, on the ward…… but these elders or seniors that are supposed to take us through the training and then groom us into this profession. They perceive us as a threat as people who are going to take their jobs at the end of the day. They say I am a diploma nurse to teach a bachelor nurse. How is that possible? In that way, we tend to miss out on many things that maybe people with experience within the profession could have taught us. That is greatly impacting us and our professional identity at the end of the day (PO2 FGD3 student).

### There is no clear scope of practice for each carder.

The participants also mentioned that whereas there is a current scope of practice for Nursing and midwifery, there is no clear scope of practice for each carder in Nursing, and this affects what the staff on the ward can teach and mentor the students.

It’s so unfortunate that we basically do the work we do in the wards is not different from what certificate nurses are doing or registered nurses; we are doing the same there’s no difference that this one did a degree, you will not stand out in any way, and that has sort of I think hindered the government from appreciating the degree nurses because there’s literally nothing new you bring on the table because we lean the pharmacology, we just administer drugs which is very sad (P08 FGD1 recent graduate).

The way the fellow people we work with, let’s say the doctors, the way they treat us. They take us to be can really make one feel not to identify ourselves as a nurse because even the smallest thing you would think of, a doctor, as long as S/he knows that this is a nurse’s work, they will have to look for you wherever you are to just come and do the small task (P09 FGD1 recent graduate).

### Low esteem among nurses and midwives

The participants also acknowledged that nurses and midwives view themselves as worthless, useless and unknowledgeable. As a result, they might feel that they cannot offer appropriate care and later lone mentor the students under them.

Our deployment as a country because we cannot promote nursing at higher levels of education, and we cannot deploy them; we cannot even set a clear scope of practice for them. I just got discouraged. I don’t know whether they are preparing us to work for this country (P01 FGD2 Recent graduate).

We don’t believe in ourselves; we always see ourselves as the weaker partners, which sometimes makes us miss out. You know, sometimes in places where we must talk, we remain silent, just (P02 FGD1 Recent graduate).

Low self-esteem cuts across the whole profession; for some reason most nurses believe that we are the weaker profession, and so that hinders a lot of things right away from communication, leadership, and all other things (P02 FGD1 Recent graduate).

### Media

The participants acknowledged that the media is good; however, instead of showing the good side of nursing and midwifery, they wait for the negative news that they publicise, affecting the students and recent graduates associating with the profession.

Media affects us a lot as nurses; as we build this professional identity, we realise that the media is always putting out the bad version, the worst of what a nurse can do. And yet, there are so many other good things that nurses do. As growing nurses as future nurses, it affects us as we build this identity (P01 FGD3 student).

### Lack of policy implementation

Participants also mentioned that Policies governing Nursing exist. However, implementing these policies is poor, affecting the nurses and midwives associated with the profession. They cited that the salary scale policies are not fully implemented in the country.

Our deployment as a country because we cannot promote nursing at higher levels of education, and we cannot deploy them; we cannot even set a clear scope of practice for them. I just got discouraged. I don’t know whether they are preparing us to work for this country (P01 FGD2 Recent graduate).

You find someone who has done critical care and is hired as a pediatric nurse and not even in an ICU for paediatrics but somewhere else, which completely detaches them from what they have specialised in (P01 FGD2 Recent graduate).

## Discussion

This study explored the understanding of professional identity and barriers to professional identity formation among student nurses, midwives, and recent graduates. The participants reported understanding of PI in nursing and mentioned that these are principles, characteristics and values, competencies, ethics and code of conduct, sense of belonging and professionalism that define the nursing profession and practice. Barriers to the formation of PI included the education theme (nursing educators not working in clinical settings and inadequate clinical mentoring) and health service delivery theme (high workload, lack of interprofessional collaboration, many different professional groups, no clear scope of practice for the different professional careers, Low esteem among nurses and midwives, media and lack of policy implementation).

Understanding professional identity influences how nurses and midwives perceive, explain, present and conduct. This study explored the understanding of the ”participants’ professional identity. Most of the participants perceived characteristics and values, ethics, and code of conduct within the concept of Professional identity as very important. Nursing ethics are deeply rooted in the nursing profession. These serve as moral compasses to promote a high level of care or ethical standards for those entering the nursing profession, affirming the responsibility of being a nurse and midwife. This is in line with the American Association of Colleges of Nursing (2021), which states that ethics is core to the nursing practice, and this guides the person’s behaviour. These are commonly accepted principles like autonomy, beneficence, non-maleficence and justice ( ANA 2012; ACNM,2015; ACNM2015, 1CN, 2012). Professional nursing values and ethics serve as principles of human dignity, integrity, altruism and justice that form a foundation of professional practice that is important for the nursing profession.

Nurses and midwives having the highest level of knowledge will influence how they do their work efficiently, which will result in the well-being of the patients and improve the provision of medical and nursing care but also increase the satisfaction of Nurses and midwives with their work. In this study, the participants agreed that professional identity means that you should be competent in what you do, which is achieved through nursing education and clinical practice. This aligns with The conceptual framework developed by the International Society for Professional Identity in Nursing (ISPIN), which presents the current thoughts on professional identity. This framework states that professional identity must have a component of Professional comportment, which is seen as a nurse’s professional behaviour demonstrated through words, actions, and presence’ (ISPIN, 2020) and knowledge, which is the analysis and application of information derived from nursing and other disciplines’ experiences, critical reflection and scientific evidence. According to Idczak(2007), students stated that knowledge was among the most important criteria for professional development and identity of the job.

Encouraging Professional identity(PI) in nursing education, clinical practice, and regulation can transform the working environment that supports nurses’ and midwives’ well-being, prevent burnout, mental and physical stress, and Job satisfaction and retention. In this study, we explored the barriers to PI formation. The participants stated that nurse educators not following them to the clinical area affected their PI formation, and they felt that the mentorship was inadequate. Nursing faculty should always aspire to role model professional behaviour in a variety of ways and provide nursing students with multiple opportunities to hone their professional identity [14], Professional mentoring of students is important because mentoring models professional behaviours [14]. This is in line with a study done in China, which found that Professional identity and clinical teaching behaviour were negatively related to transition shock. A better sense of identity and supportive clinical teaching were keys to a smoother journey from new to experienced nurses [15]. Faculty need informal and formal mentoring of nursing and midwifery students to create a professional identity congruent with the competencies outlined by the curriculum and practice.

Nursing workload affects the time a nurse can allot to various tasks. Under a heavy workload, nurses may not have sufficient time to perform tasks that can directly affect patient safety [16]. In this study, the students felt that the clinical nurses and midwives had a high workload, which affected the care that was given to the client and these health workers are supposed to teach them in the process. This affected the students’ learning, and they questioned whether this was the right profession to join. This is in line with a study that found that lack of time, dual responsibility, heavy workload, personality, and attitude may negatively impact the mentoring process and eventually fail to foster professional identity among students. [17]. Another study found that practitioner workload may impact the student experience due to challenges in sufficient time to provide support. [18]. There is a need for clinical health workers to pay attention to improving the professional attraction to nursing programs by improving the understanding of the profession and reducing work intensity through delegation.

Interprofessional and interprofessional collaboration improves the quality of care. In this study, the students mentioned that interprofessional and intra-professional collaboration affected their professional identity. Most of the other professionals did not understand the roles of other carders, and they gave them functions that were not in line with their carders. However, this is contrary to a study that showed that Interprofessional identity positively affects congruent interprofessional behaviours [19]. It is important to have good interprofessional collaboration because it helps to train different disciplines to learn how to work together and recognise the value of different skill sets and efforts that enhance the workplace. One study found a collaborative work environment to improve conflict management, confidence, and innovation while lowering emotional exhaustion. This benefits healthcare workers by reducing workload and increasing job satisfaction.

The scope of nursing practice is the range of roles, functions, responsibilities and activities a registered nurse is educated, competent and has authority to perform. The profession must be able to clearly articulate its practice parameters to ensure that nursing practice can accommodate and respond to the current needs of society. In this study, the students mentioned that not having a clear scope of practice for each carder affects professional identity formation. These roles need to be clearly stated to facilitate professional identity. The absence of a defined scope of practice for the different health workers may negatively impact the quality of care and patient safety (Afzal et al., 2018), consequently affecting professional identity formulation. There is a need for the regulatory bodies to clearly state the scope of practice for each carder in nursing. This will boost professional identity formation.

Self-esteem is an important factor contributing to one’s subjective feelings of value as a professional and may play a central, transformative role in developing professional values and identity [20]. In this study, the participants said that the low esteem of nurses and midwives affected their professional identity formation. There is a need for the patients, colleagues, and families to acknowledge the work of nurses and midwives. This makes nurses feel valued as persons and enables them to see the value of their work, eventually improving their professional identity.

The media play an important role in shaping ”nurses’ professional identities. They can influence how the public perceives nurses, how they see themselves and how they interact with patients and colleagues. Although the media can have many positive effects on professional identity, they can also have negative consequences, especially when they perpetuate stereotypes, promote unrealistic expectations or portray the profession negatively. In this study, participants mentioned that sometimes, the media only portrays the negative aspects that affect the profession. This is in line with this study, which states that the image of nursing depends on how nurses themselves and others (the public) perceive nursing [21]. This public image is predominantly based on misconceptions and stereotypes in distorted images of nurses in the media [21].

In conclusion, the participants had a fair understanding of professional identity and faced several challenges in professional identity formation. These challenges ranged from educators and health service delivery (clinical area). There is a need to streamline the scope of practice and enhance clinical mentorship and engagement of Leadership in Nursing in developing PI among students.

### Strength and limitation

To our knowledge, this study is new as it is the first to explore the understanding of professional identity and barriers to professional formation in Uganda. Participants provided rich and varied descriptions of their experiences, providing a comprehensive insight into clinical learning. Given the data collection and analysis quality, the study is highly rigorous, and the findings are credible. Despite these strengths, the findings may be limited to the student’s perspective but are transferable to similar contexts.

## Data Availability

The datasets used and/or analysed during the current study are available from the corresponding author on reasonable request

## Abbreviations

MRRH: Mbarara Regional Referral hospital
MNRH: Mulago National Refferal Hospitla
PI: Professional identity
UNCST: Uganda National Council for Science and Technology
